# Diffuse Large B-Cell Lymphoma Presenting With Hypercalcemia

**DOI:** 10.7759/cureus.73642

**Published:** 2024-11-13

**Authors:** Yasser Hegazy, Mike Chung, Nicholas Zamora, Heng-Tien Aaron Lee, Muhammad Ghallab

**Affiliations:** 1 Internal Medicine, Icahn School of Medicine at Mount Sinai, Queens Hospital Center, New York, USA; 2 Internal Medicine, The New York Institute of Technology College of Osteopathic Medicine, New York, USA; 3 Internal Medicine, St. George's University School of Medicine, True Blue, GRD

**Keywords:** 1`25-dihydroxy vitamin d, diffuse large b cell lymphoma (dlbcl), hypercalcemia, malignancy-related hypercalcemia, non-hodgkin's lymphoma

## Abstract

Hypercalcemia is a common metabolic complication associated with malignancies, particularly in solid tumors, such as lung and breast cancers. However, its occurrence in hematologic malignancies, including diffuse large B-cell lymphoma (DLBCL), is rare. The pathophysiology of hypercalcemia in lymphomas is often related to the secretion of parathyroid hormone (PTH)-related peptide (PTHrP). However, an alternative and less common mechanism involves the ectopic overproduction of 1,25-dihydroxyvitamin D (1,25(OH)2D), which has been reported in some cases of lymphoma-associated hypercalcemia. In this case report, we present a 60-year-old female who was incidentally found to have hypercalcemia during the evaluation of nonspecific symptoms, ultimately leading to the diagnosis of DLBCL with liver and splenic involvement. The patient presented to the emergency department with symptoms of shortness of breath, palpitations, insomnia, polydipsia, and polyuria. Laboratory evaluation revealed a critical calcium level of 12.5 mg/dL, along with suppressed PTH levels and elevated 1,25(OH)2D levels, suggesting an ectopic source of 1-alpha-hydroxylase activity. Imaging revealed hepatic and splenic masses, and a liver biopsy confirmed the diagnosis of DLBCL. Further evaluation ruled out more common causes of hypercalcemia, such as osteolytic metastases and PTHrP-related mechanisms, reinforcing the likelihood that the patient’s hypercalcemia was due to ectopic 1,25(OH)2D production. This case underscores the complexity of hypercalcemia in patients with DLBCL, particularly when uncommon mechanisms such as ectopic 1,25(OH)2D synthesis are involved. While the exact etiology of hypercalcemia in DLBCL remains incompletely understood, it is emerging as a potential biomarker for poor prognosis. Studies suggest that hypercalcemia in DLBCL may correlate with aggressive disease features, shorter diagnosis-to-treatment intervals, and worse overall outcomes. In this case, the patient’s hypercalcemia may reflect the biological aggressiveness of her disease, with a relatively short interval to treatment after diagnosis. Further research is necessary to better understand the prognostic significance of hypercalcemia in DLBCL and its underlying mechanisms.

## Introduction

Hypercalcemia is a well-recognized metabolic complication in oncology, with a prevalence of 3%-30% among cancer patients [1]. It is most commonly associated with solid tumors, particularly lung and breast cancers, where the incidence of hypercalcemia ranges from 24% to 28% [2]. In hematologic malignancies, hypercalcemia is frequently observed in multiple myeloma, primarily due to local osteolysis [3]. Hypercalcemia also sometimes occurs in patients with leukemia or malignant lymphomas but to a lesser extent than in those with multiple myeloma [4]. The reported prevalence of hypercalcemia in non-Hodgkin's lymphoma is 1.3%-7.4%, while the prevalence of hypercalcemia in diffuse large B cell lymphoma (DLBCL) at diagnosis is unknown [5]. The pathophysiology of hypercalcemia in lymphomas is often attributed to the secretion of parathyroid hormone-related peptide (PTHrP), a mechanism commonly seen in human T-cell lymphotropic retrovirus type 1-associated lymphomas and certain non-Hodgkin's lymphomas [4,6]. PTHrP-induced hypercalcemia is typically characterized by low levels of 1,25-dihydroxyvitamin D (1,25(OH)2D) and metabolic alkalosis. However, a less common mechanism involves the ectopic overproduction of 1,25(OH)2D, which has been reported in some cases of lymphoma-associated hypercalcemia [7].

This case report presents a 60-year-old female with incidental hypercalcemia, discovered during the evaluation of nonspecific symptoms, which led to the diagnosis of DLBCL with hepatic and splenic involvement. The hypercalcemia in this patient was most likely driven by increased ectopic 1-alpha-hydroxylase activity, causing ectopic production of 1,25(OH)2D, a less frequent but recognized mechanism in lymphoma-associated hypercalcemia.

## Case presentation

A 60-year-old female, with a past medical history of hypertension, obesity, and prediabetes, presented to the emergency department with complaints of shortness of breath, palpitations, insomnia, polydipsia, and polyuria. The patient reported progressive exertional dyspnea, which was accompanied by palpitations and limited exercise tolerance to walking a single block. Additionally, she described a daily intake of more than 10 bottles of water and excessive urination, along with an unintentional weight loss of unknown magnitude. The patient consumed alcohol occasionally but denied any history of smoking.

On presentation, the patient's vital signs were notable for tachycardia, with a heart rate of 110 beats per minute. A physical examination revealed no significant abnormalities. Initial laboratory findings were remarkable for hypercalcemia. The complete blood count (CBC) indicated a white blood cell count of 10.43, with mildly elevated monocytes at 12.4% and immature granulocytes at 2.3%, along with mild normocytic normochromic anemia (hemoglobin of 10.9) and a normal platelet count at 320. Additional laboratory abnormalities included an elevated D-dimer, elevated alkaline phosphatase, elevated aspartate aminotransferase (AST), and gamma-glutamyl transferase (GGT). Parathyroid hormone (PTH) was suppressed, and angiotensin-converting enzyme (ACE) was elevated, raising the suspicion of sarcoidosis. The patient also had an elevated lactate (Table 1).

**Table 1:** Summary of laboratory test results

Laboratory Test	Result	Normal Range
Calcium	12.5 mg/dL	8.6–10.2 mg/dL
D-dimer	2,699 ng/mL	<500 ng/mL
Alkaline Phosphatase (ALK Phos)	717 U/L	44–147 U/L
Aspartate Aminotransferase (AST)	241 U/L	10–40 U/L
Gamma-Glutamyl Transferase (GGT)	857 U/L	9–48 U/L
Parathyroid Hormone (PTH)	2 pg/mL	10–65 pg/mL
Parathyroid Hormone-Related Protein (PTHrP)	<2.0 pmol/L	<2.0 pmol/L
Vitamin D25 Hydroxy	10.8 ng/mL	30–100 ng/mL
Vitamin D1,25 Dihydroxy	196.8 pg/mL	18–72 pg/mL
Angiotensin-Converting Enzyme (ACE)	100 U/L	8–52 U/L
Lactate	3.6 mmol/L	0.5–2.2 mmol/L

The patient was admitted for further evaluation and management of symptomatic hypercalcemia. PTH-related protein (PTHrP) was within normal limits, while vitamin D25 hydroxy was low, and vitamin D1,25 dihydroxy was elevated. A viral hepatitis panel was negative, except for a positive hepatitis B surface antibody, likely due to prior vaccination (Table 1).

**Figure 1:**
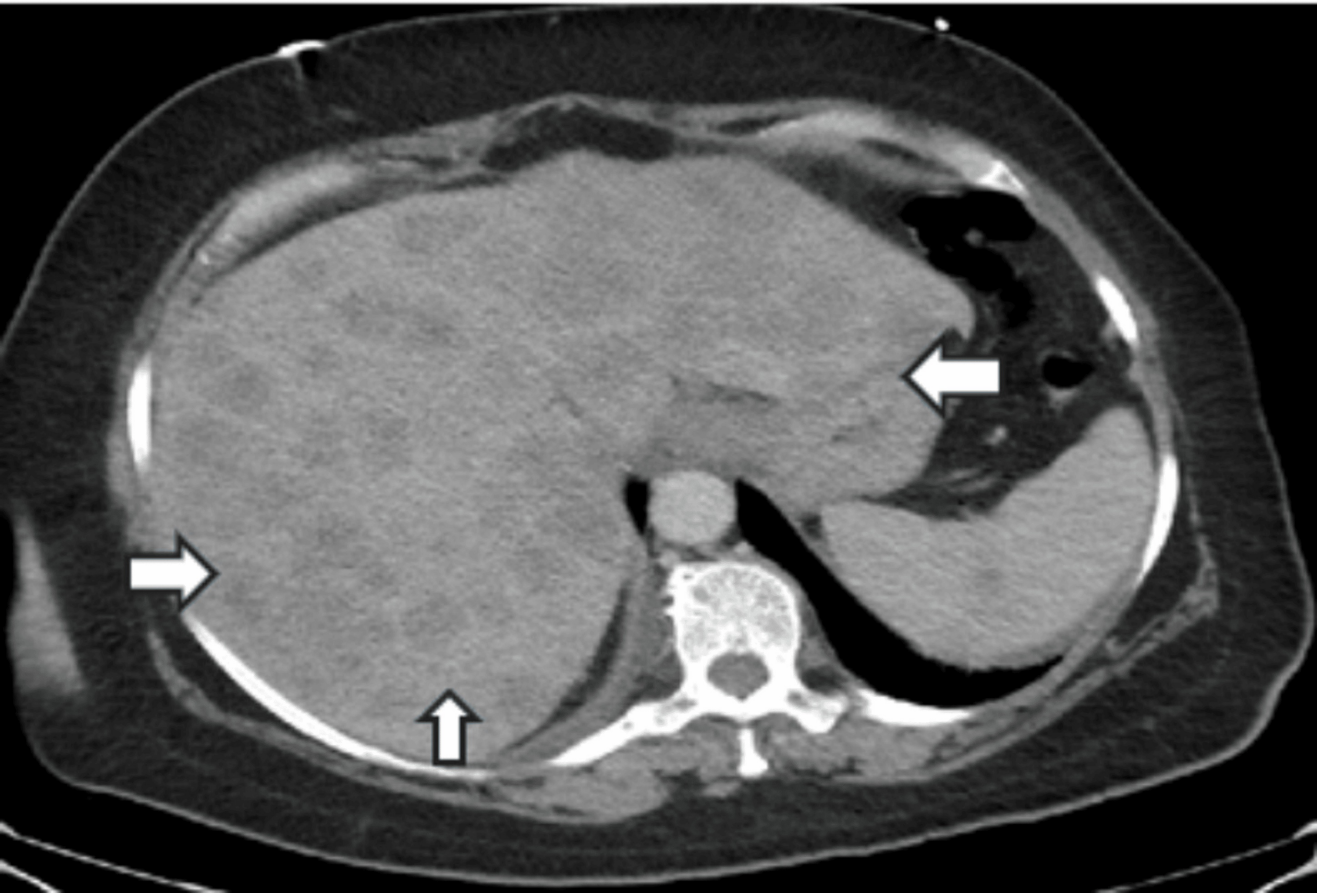
The arrows indicate the enlarged liver showing bilobar poorly enhanced lesions

**Figure 2:**
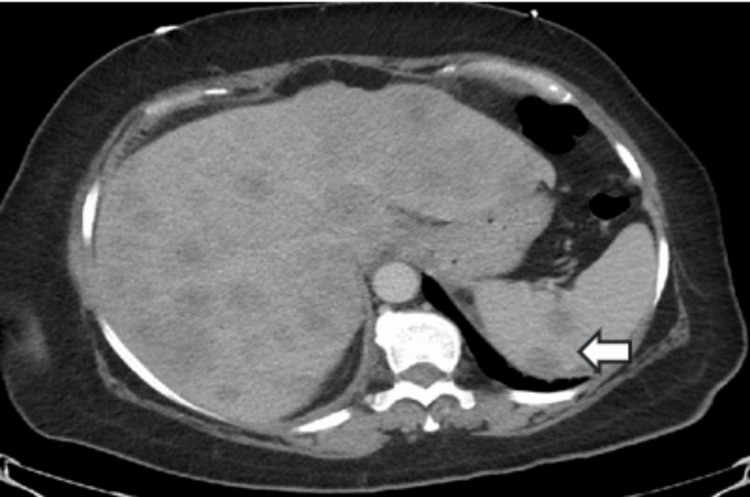
The arrow indicates splenic hypodense lesions

Computed tomography (CT) angiography excluded a pulmonary embolism but revealed enlarged pericardial and right internal mammary lymphadenopathy, raising concerns for malignancy. Additional findings included hepatic fibrosis and a 7 mm right upper lobe pulmonary nodule. A CT of the abdomen and pelvis showed an enlarged liver with bilobar and poorly enhanced lesions (Figure 1), suspicious for metastatic disease. The spleen contained two hypodense lesions, the largest measuring 2.3 cm in diameter (Figure 2).

An image-guided biopsy of the liver mass confirmed the diagnosis of diffuse large B-cell lymphoma (DLBCL). The patient was started on zoledronic acid for the management of hypercalcemia, with subsequent improvement in calcium levels.

## Discussion

This case presents a 60-year-old female with hypercalcemia (12.5 mg/dL) as an incidental finding, ultimately leading to the diagnosis of aggressive DLBCL with multiple liver and splenic masses. Hypercalcemia is a common metabolic complication of malignancy, and several mechanisms have been proposed to explain its occurrence. The most frequent mechanism is malignancy-associated hypercalcemia mediated by the production of PTHrP [4,6]. Although several cases of DLBCL presenting with elevated PTHrP have been reported, this mechanism does not explain our patient's condition, as her PTHrP levels were within normal limits (<2 pmol/L).

Ectopic production of PTH is another possible cause of malignancy-associated hypercalcemia [6]; however, the patient's suppressed PTH level (<2 pg/mL) makes this hypothesis unlikely. Another plausible explanation is osteolytic metastases, leading to excessive calcium release from bone [4]. However, a PET CT scan reveals only degenerative changes in the spine with no evidence of suspicion of lytic or blastic bone lesions.

The most likely explanation for hypercalcemia in this patient is increased ectopic 1-alpha-hydroxylase activity causing ectopic production of 1,25(OH)2D [4,6]. Elevated 1,25(OH)2D stimulates intestinal and renal calcium absorption, contributing to hypercalcemia [8]. The patient’s elevated 1,25(OH)2D (196.8 pg/mL) and decreased 25-hydroxyvitamin D (10.8 ng/mL) are consistent with this mechanism, possibly due to increased ectopic 1-alpha-hydroxylase activity, which converts 25(OH)D to 1,25(OH)2D. It is important to note that hypercalcemia in sarcoidosis shares a similar pathophysiological mechanism. Given the presence of innumerable masses in the liver and spleen on imaging, extrapulmonary sarcoidosis was considered a differential diagnosis; however, biopsy findings confirmed metastatic malignancy, ruling out sarcoidosis.

While the precise etiology of hypercalcemia in patients with DLBCL remains incompletely understood, it may occur through multiple mechanisms. Importantly, hypercalcemia has emerged as a potential biomarker for DLBCL prognosis. Gauchy et al. conducted a retrospective study from 2006 to 2018, examining the prevalence and prognostic implications of hypercalcemia in patients with DLBCL [9]. Their findings revealed a 24% prevalence of hypercalcemia at the time of DLBCL diagnosis, and this condition was strongly associated with high-risk features such as International Prognostic Index (IPI) components, B symptoms, elevated β2-microglobulin, and abnormal hemoglobin and albumin levels. Moreover, hypercalcemic patients had a significantly shorter diagnosis-to-treatment interval (DTI), which has been linked to worse overall and progression-free survival [10,11]. Gauchy et al.’s study demonstrated that hypercalcemia is a predictor of poorer event-free survival (EFS24) (HR = 1.66; 95% CI: 1.08-2.54), shorter progression-free survival (PFS) (P = 0.0059), and overall survival (OS) (P = 0.0003) [9]. These findings suggest that hypercalcemia may indicate the biological aggressiveness of DLBCL [9]. In our case, the patient commenced R-CHOP chemotherapy 12 days after diagnosis, a relatively short DTI, which could imply a poor prognosis. Further research is needed to solidify the role of hypercalcemia as a prognostic biomarker in DLBCL.

## Conclusions

This case highlights the complex interplay of metabolic abnormalities in a patient with DLBCL presenting with hypercalcemia. The patient's hypercalcemia was likely driven by increased ectopic 1,25(OH)2D production, a mechanism frequently seen in both malignancies and granulomatous diseases. The presence of hypercalcemia has been established as a negative prognostic indicator in DLBCL, correlating with aggressive disease features and poor clinical outcomes. In our patient, a short diagnosis-to-treatment interval further raises concern for an unfavorable prognosis.

## References

[1] Ogawa M, Morikawa M, Kobatake M (2022). Hypercalcemia associated with the ectopic expression of 25-hydroxyvitamin D3-1α-hydroxylase in diffuse large B-cell lymphoma. Intern Med.

[2] Santarpia L, Koch CA, Sarlis NJ (2010). Hypercalcemia in cancer patients: pathobiology and management. Horm Metab Res.

[3] Gastanaga VM, Schwartzberg LS, Jain RK (2016). Prevalence of hypercalcemia among cancer patients in the United States. Cancer Med.

[4] Stewart AF (2005). Clinical practice. Hypercalcemia associated with cancer. N Engl J Med.

[5] Abadi U, Peled L, Gurion R, Rotman-Pikielny P, Raanani P, Ellis MH, Rozovski U (2019). Prevalence and clinical significance of hypercalcemia at diagnosis in diffuse large B-cell lymphoma. Leuk Lymphoma.

[6] Mirrakhimov AE (2015). Hypercalcemia of malignancy: an update on pathogenesis and management. N Am J Med Sci.

[7] Breslau NA, McGuire JL, Zerwekh JE, Frenkel EP, Pak CY (1984). Hypercalcemia associated with increased serum calcitriol levels in three patients with lymphoma. Ann Intern Med.

[8] DeLuca HF (1986). The metabolism and functions of vitamin D. Adv Exp Med Biol.

[9] Gauchy AC, Kanagaratnam L, Quinquenel A (2020). Hypercalcemia at diagnosis of diffuse large B-cell lymphoma is not uncommon and is associated with high-risk features and a short diagnosis-to-treatment interval. Hematol Oncol.

[10] Maurer MJ, Ghesquières H, Link BK (2018). Diagnosis-to-treatment interval is an important clinical factor in newly diagnosed diffuse large B-cell lymphoma and has implication for bias in clinical trials. J Clin Oncol.

[11] Yoshida M, Nakaya Y, Shimizu K (2021). Importance of diagnosis-to-treatment interval in newly diagnosed patients with diffuse large B-cell lymphoma. Sci Rep.

